# Practical Observations in Surgery and Morbid Anatomy

**Published:** 1817-09

**Authors:** 


### *Practical Observations in Surgery and Morbid Anatomy.* By John Howship, M. R. C. S. &c. &c.

(Continued from our last Number, p. 237.)

The following case ought to prove a warning to those who are in the habit of striking boys on the head with rulers, when at school.

Case I. A young gentleman, 12 years of age, received a rap with the edge of a flat ruler because he was dull in learning. A small wound was the consequence, which could not be healed till after a period of six years. It then closed, and he soon perceived that his eye-sight was failing, to which were added epileptic fits that returned every day. In this state, he consulted Dr. Lettsom and Mr. Heaviside. The cicatrix of the old wound exhibited nothing unusual. It was proposed that a portion of the bone should be removed by the trephine, and the operation was performed. Some serous fluid and blood escaped from between the skull and dura mater. The membrane, however, had not lost its healthy colour. Next day, the pupils of the eyes recovered their natural sensibility. The blindness remained absolute as before. A slow fever now supervened, and on the third day after the operation he was carried off by a severe convulsion.

*Dissection.* The cranium and dura mater were every where sound. Opposite the seat of the original wound, the pia mater had evidently suffered from chronic inflammation. The brain here was found indurated to a considerable degree, which induration extended itself to the whole of the middle lobe of the cerebrum, down to the basis cranii. There were no other morbid appearances.

Case II. *Habitual Eruption driven in upon the Brain.* Miss C. T. a young lady aged 24, had been for several years subject to an eruptive complaint upon the face, which was frequently very troublesome. Her menstrual health was often deficient, and when this was the case, her face usually became heated and irritable; largish pimples, of a dull red colour, made their appearance, and the irritability being hardly supportable, these pimples were sometimes scratched, and would bleed.

"For so unpleasant a complaint she was naturally anxious to find a cure. It was mentioned to me repeatedly; but as it had been ascertained by experiment, that neither bark nor steel agreed with her, I advised her to bear with it, but by no means to use any local application with a view to its removal. This opinion satisfied her for some time, but in June, 1813, she was prevailed upon by a female friend, to apply a lotion to her face, which certainly answered its purpose, for it cleared the face presently: but as the heat left the cheeks she began to feel uneasiness in the head; and by the time the eruption was pretty well removed from the skin, she complained of a tremendous sense of fulness and severe pain in her head; soon after which she became delirious. Bleeding, blistering, and much attention were necessary to relieve the severity of this attack, but the object which, of course, was to bring back the eruption, if possible, to the face, was not accomplished in less than three months, during which period she continued to suffer from extreme head-ache." p. 133.

Mr. H. next relates the case of a man, in whom suppressed perspiration from the feet was followed by symptoms of effusion on the brain.

Case III. J. P. 77 years of age, had long been subject to excessive perspiration on the lower extremities; in other respects he was well. He was advised to apply fresh dock leaves to his feet soles. He first felt a tingling sensation and irritation of the skin; in half an hour, he experienced great uneasiness and pain in the head, especially over the eyes; and in less than an hour he was nearly blind! He was taken to St. George's Infirmary, where he remained the whole of the day and the night, labouring under intense head-ache and indistinctness of vision. He had no constitutional disorder. Next day the pupil became insensible to light. A blister behind each ear, and to the soles of the feet. Calomel in small doses with a view of affecting the system. As soon as the blisters became painful, the head-ache and blindness were relieved. The blistered surfaces were kept open; and the feet were ordered to be frequently immersed in warm water, and afterwards wrapt in flannel. Under this treatment the patient was gradually restored to health, the process being accelerated by a mercurial ptyalism.

*Affections of the Larynx.* Of all cervical affections, those of the air tube are the most important, since fatal obstructions advance here with insidious steps at first, but rapid marches ultimately. The functions and structure of the larynx are very complicated. Every modulation of the voice is an effort of volition regulating a number of minute muscles moving the parts within the laryngeal cavity. Hence it is evident, that a very slight derangement in parts so constituted must produce much mischief. Dissection shews, that in laryngeal inflammation, it is by no means necessary that *ulceration* take place, or that a secretion of purulent or coagulable matter be thrown out into the cavity of the larynx, in order that suffocation may be induced. A tumid state of the parts from œdema produced by an effusion of serous fluid into the cellular texture, is all that is frequently found *post mortem*. The varieties of inflammatory affection to which these parts are liable have not been accurately defined; but many valuable cases have lately been laid before the public, elucidatory of the subject. Such are the rapidity and fatality of tracheal inflammation, that any degree of *dyspnœa* should always excite serious apprehensions in our minds, and induce us to adopt decisive measures, before the favourable moment for preventing the complete establishment of the disease elapses. Every means of lowering the circulation should be instantly put in force, with a view to diminish, first, quantity, and then action. General and local bleeding must pave the way for blisters, diaphoretics, and antispasmodics. When dyspnœa has risen to distressing anxiety—risk of suffocation, with occasional delirium, then bronchotomy is our only resource. Mr. Howship does not think it material at what point this is performed. Spasm of the muscles of the glottis, is Mr. H. thinks, much less frequently concerned in the fatal event, than was supposed by Dr. Cullen.

Case IV. *Abscess in the Cavity of the Larynx.*

"This patient was a soldier, about thirty years of age; he had complained, in the first instance, of a very painful swelling on the muscular part of the left thigh. The tumour was hard, heated, and red, much resembling a phlegmonous inflammation Added to this, he was feverish, and could get no rest at night.

"By sedative applications and saline medicines, the inflammation was nearly dispersed at the end of a week, when a second tumour, precisely resembling the first, arose upon the fleshy part of the left fore-arm, and was attended with severe pain. This swelling was poulticed, fomented, and opened, and about four ounces of matter discharged. The abscess suppurated copiously for a week. There now commenced a violent swelling and inflammation upon the back part of the right hand. This was fomented for three days, during which time he had not the least power of moving his fingers. The symptoms then began to abate, and by the end of a week from its commencement, this swelling was entirely dispersed.

"The ulceration of the fore-arm was evidently connected with the tendinous parts of the flexor muscles; and portions of the fascia, with sloughy fragments of tendon, were repeatedly found in the opening of the wound, and were removed. A probe readily passed upward and downward in the diseased muscular and cellular structure. When it had been open about a week, the cavity threw up florid granulations. It had discharged ten days, when it was observed that the quantity of matter formed, had suddenly diminished. In two days more, the sore was absolutely dried up, so that the applications were found the next morning unsoiled. On the same day, he said he had an uncomfortable sensation about his throat. This he again mentioned a few days afterwards, and said it was getting worse. Respiration was now somewhat impeded, not as if dependent on oppression in the chest, but as if from some obstruction to the free transmission of the air into the lungs.

"Towards evening his articulation became affected, his breathing difficult to the most distressing degree, and his countenance full of wild alarm. The difficulty of respiration continued to increase rapidly, in consequence of which, at midnight, a consultation was called. He was supported in bed, and took æthereal and antispasmodic mixtures, and had a very large blister laid to the chest; about six in the morning he died.

*Examination* "On dissection, no particular secretion of mucus was found in the larynx, the trachea, or its ramifications. The whole of the mischief was confined to the thyroid cartilage, the cavity of which had become so much narrower than usual, as to have been the evident cause of suffocation.

"The cartilage, divided on one side, was laid open, and the parts carefully washed, when the cellular substance connecting the mucous membrane to the inner surface of the cartilage was found inflamed, diseased, and very much increased in thickness. That portion of the membrane lining the thyroid cartilage immediately below the arytenoide cartilages, was astonishingly thickened, forming on each side the cavity, a spongy, elastic cushion, projecting in such a manner, that the two opposite surfaces were found as nearly as possible in perfect contact.

"The surface of the diseased parts was of a firm texture, and dull reddish colour; and when cut into and pressed, numerous globules of thick purulent matter issued forth from the diseased structure. In the colour, the feel, and the manner in which suppuration had taken place in this instance, the disease bore a striking analogy to carbuncle, only it was upon a smaller scale."—p. 159.

*Affections of the Heart.* Although our author has seen comparatively few diseases of the heart, yet he is convinced that any thing like accuracy of diagnosis in morbid affections of this organ, during life, is, in every case, extremely difficult—in fact, that "all opinion must rest upon conjecture." This conviction, says Mr. H. is not derived from his own limited experience in practice, but rather from the number of bodies that he has had an opportunity of examining after death; in some of which, physicians of the highest reputation had believed the heart free from disease, when dissection proved the contrary; while in others, almost every physician of character in London had decided on cardiac disease, while *post mortem* inspection shewed the heart perfectly sound. One instance of this kind was a lady, about the turn of life, a robust woman, whose body Mr. Howship was requested to open, with the view of preserving the heart. But this viscus was in every respect sound; and the only morbid appearances were, a small calculus in the gall bladder, and a trifling effusion of serum in the cavity of the abdomen.

The truth is, that *functional* disorder of the heart frequently simulates *organic* derangement; and the *symptoms* of this last are so frequently evinced at a *distance* from the central organ of circulation, that we have repeatedly been mistaken, and times out of number mortified at the bad success of our diagnostic knowledge; yet still we do not go so far as Mr. Howship, in declaring that "all opinion must rest on conjecture." We have been wrong respecting the nature of the affection, but we have also been often right; and we still think that the class of cardiac diseases is one of the highest importance to be studied most carefully by every practitioner in the healing art. We shall here condense a very interesting case, illustrating the difficulty of discriminating organic changes in the heart.

Case V. J. King, a boy 12 years of age, became poorly, without apparent cause, in January 1811. He complained of pain in his back, and under his heart. He sometimes said his "heart ached," but tea or other warm fluid gave him temporary relief. One day in February, when his mother entered the room, she found the child standing up, talking wildly in a delirium, and declaring the room to be full of people. Next day he was better, but unable to go to his usual work. Towards evening, he fell into a raging fever and ungovernable delirium, running about the room, and destroying every thing that came in his way. He was seen by an apothecary. He complained, though light-headed, that his "heart ached" When laid in bed, he requested to be supported with pillows. Considerable fever and oppression at the chest during the night. The treatment prescribed not giving much relief, two very large blisters were laid on the sides of the thorax, from which the child experienced so much benefit, that he requested to have more applied. The fever, thirst, and difficulty of breathing, however, still continued through March and April, and at length he could only make himself understood by signs. A physician now visited him. The child described his complaint as being in the chest and about his heart. The fever at this time was variable both in period and intensity. In July, his legs began to swell, and this tendency increased. The dyspnœa was now attended with some cough. During the last month of his life he could not lie down at all. The state of the bowels was variable, but generally confined. The latter end of August put an end to his miserable existence by a convulsive fit of coughing.

*Sectio cadaveris.* Abdomen externally tense and large; body generally emaciated. The pericardium externally felt full, and was much larger in volume than natural, filling at least three-fourths of the whole cavity of the chest. Lungs universally adherent to the ribs and diaphragm, with the exception of some spaces, which were filled with serum. The pericardium adhered intimately to the heart. The latter enormously enlarged. The internal organization of the heart, however, was not deranged. The liver had suffered inflammation, and was adherent to the diaphragm, and in these adhesions were several capsules filled with yellow serum. We could make some objections to Mr. Howship's reasonings on the cause of this enlargment of the heart, but think it would appear captious. The facts themselves are valuable.

Case VI. In February 1813, Mr. H. accidentally saw a girl, 13 years of age, sitting supported in her mother's arms. Her countenance was livid and bloated. She was perfectly sensible, but without power of motion or utterance. No pulse at the wrists; but on laying the hand on the region of the heart, there was a most violent and extraordinary action of the heart, the contractions of which were extremely powerful and quick, but irregular. This attack was what the mother called a fit, to which the child had been subject for some years past. About six years before, she had been for a short time hot and feverish, and ever since had laboured occasionally under paroxysms similar to the above. In proportion as these fits of palpitation became more frequent and violent, the change of colour during the attack became more remarkable. In these, the body was nearly cold, and perfectly livid, with every character of complete strangulation, notwithstanding the extreme violence of action in the heart. This child was naturally very susceptible of the slightest impression. If chid at school, she became low spirited and unhappy for the day, and her complaints were always the worse for it. She never seemed so happy as when silent and inactive. The appetite and powers of digestion were unimpaired. She died in one of these paroxysms.

*Examination.* Pericardium closely adherent to the heart. The latter prodigiously enlarged; but unequally so. The right auricle and ventricle were of the natural size; the left cavities enormously dilated, and containing at least a pound of black grumous blood. The dilatation was active. The auricular valve of the left ventricle had become thickened in its structure, and quite incapable of performing its office; in short, the auricle and ventricle were thrown into one.

CASE VII. *Singular Disease of the Lungs.* "In August 1803, I was desired to examine the body of a Mrs. Roberts, a lady of a delicate habit of body, who died at the age of 27 years. The following were the leading circumstances of her previous history.

"Some months before her death, she had been at an assembly, and after dancing till she was very much fatigued, had walked home, and got wet through. A cold, with cough, was the consequence. Medicine and nursing soon appeared to relieve the cold, but the cough remained, and proved exceedingly violent and obstinate, although unconnected with any material degree of pain in the chest. The cough continuing, her respiration by degrees became difficult and oppressed, and this change became every day more urgent. She frequently mentioned to those around her, a most distressing sensation, as if the action of the heart was about to cease. She sometimes also suffered from a violent palpitation; and from the repetition of these attacks, she got into the habit of laying her hand often to her breast. As her complaint continued, respiration became more oppressed; and from her own feelings she was persuaded that her heart was not situated where it used to be. She said she could not feel it beat as before, not even if she laid her hand upon her breast. The cough was still very troublesome.

"Some time after this, she thought she felt her heart beating on the right side instead of the left; and, on examination, the action of the heart was distinctly perceived high up on the right side of the chest.

"This curious circumstance became more evident as the disease advanced, so that latterly the motions of the heart could be distinctly seen through the soft parts between the ribs upon the right side.

"One morning after sitting up in bed, and conversing with her attendant very cheerfully on various subjects, she desired some milk. This was brought, and she drank some. The nurse soon afterward wanting something in the next room, left her a minute or two, and on returning, found her dead.

Toward the latter stage of the complaint, she occasionally suffered under the greatest alarm, arising from her peculiar sensations; which at some times threatened immediate suffocation, at others, a total stoppage in the action of the heart.

*Examination.* "On opening into the chest, a most singular disease was discovered. A very large, heavy, and compact tumour had formed in the midst of the left lobe of the lungs, and had pressed aside the heart, pericardium and mediastinum, to the opposite side of the chest. The commencement of this tumour had sprung apparently in the cellular parenchymæ of the lungs. On cutting through the surface of the lungs, which as the disease advanced, had formed its outer covering, the structure appeared sound, although the cavities of the air-cells must have been entirely obliterated, from the continued pressure of so large a mass of disease.

"The whole of the contents of the chest were carefully removed, when a very singular, and no less curious appearance was observed. Upon the external surface of the diseased lung, a great number of processes were pendulous from the surface of the pleura. These had more or less of a bulbous form at the extremity, but the point of attachment formed a narrow peduncle, or stalk. These processes were said to resemble hydatids, but they had not the tough consistence, nor the thin, but opaque white coat of the hydatid; and although they certainly contained a fluid, it was not aqueous, but wholly coagulable by heat, or nearly so." 205.

It is not very unusual for the constitution to relieve a plethoric state of the vascular system through the medium of the lungs, and particularly in obstructed menstruation. The following is a remarkable instance of vicarious pulmonary hæmorrhage.

Case VIII. Jane Ray, ætat. 44, stout, had menstruated regularly from the age of 16 till 20 years. At this time she was confined two months with rheumatic fever, attended by violent pain in the head, *sine delirio.* The menses were now stopped. In a few months after this she had another similar attack, and again recovered. Other attacks alternated with health, till one day that, without any premonitory symptoms, she was suddenly seized with a violent fit of coughing, and with great hazard of suffocation, threw up from her lungs, in the space of an hour, two quarts of blood. The immediate consequence of this excessive evacuation was great weakness, a strong beating at the heart, and a strange sense of fluttering in the breast: the cough ceased with the hæmorrhage. The following night she slept badly, but for three months she was particularly well and active. Another discharge of blood now took place, and lasted several hours, but was not so profuse as the first. It returned, however, every day for five or six weeks; and when the consequent debility went off, she found herself more free, lively, and capable of work than she had almost ever been. She remained some months well, and then experienced another hæmorrhage from the lungs, preceded this time by sense of oppression and fulness in the breast, with anxiety and sighing. This attack was very violent, and continued more or less for five weeks, when she gradually recovered. She now experienced an interval of twelve months good health, when the old complaint returned exactly as before, and preceded by the symptoms last described. A few months after this a pulmonary hæmorrhage of more than usual violence took place. The blood poured into the trachea in so large a stream, that it might be said literally to run out of her mouth. This attack lasted nearly six weeks, and was succeeded by twelve months health. In 1806, a heavy seizure was attended with a new symptom, a violent pain in the left side, increased during inspiration. The hæmorrhage continued six weeks, and she was between two and three months in recovering. The intervals now shortened, but the attacks were less severe. From 1807 till the latter end of 1816, these attacks became more and more violent, so that several times she was at the very brink of death. She has now reached the age of 46, and it is probable, that instead of this singular disorder terminating her life, she may live now to recover entirely from it; and whether or not, the above particulars place the occasional resources of the constitution in a very curious and highly-interesting point of view.

Case IX. The power which certain parts possess of bearing the irritation of foreign bodies, accidentally or designedly lodged in them, is not to be learnt by reasoning, but by experience. In the following case, a flat-headed iron nail, seven-eighths of an inch in length, was lodged in one of the smaller branches of the trachea, where it remained near four months, and was ultimately thrown out by coughing. The man was 65 years of age, and while working in the repair of an ornamental cieling, with two nails in his mouth, was seized with a fit of coughing, when one of the nails was thrown out of his mouth; and the other, in recovering his breath, slipt down his windpipe. This event was followed by incessant irritation, pain, and cough, which continued till the poor man was worn away to a skeleton, spitting up blood and mucous phlegm. All the Faculty consulted, pronounced his case hopeless. Prescriptions mitigated his sufferings little, and could not relieve them. The pain and all his complaints were fixed in the right lobe of the lungs, and he could now, as at the first instant, cover the exact spot with his hand. Spitting of blood continued to recur at intervals, and the poor fellow was consigned to certain death. At length, on the 12th of August, 1804, during a violent fit of coughing, he threw up something with force against the roof of his mouth, mixed with blood. He spat it into his hand, and found it to be the identical nail that had slipped down the trachea so long before. Twelve years have now elapsed since this event took place, and the man has enjoyed pretty good health, subject, however, to occasional spitting of blood, and a painful sensation in the spot where the nail had been lodged.

Case X. *Singular Abdominal Disease.* A youth, ætat. 17, had, during the last twelve months, been weak and complaining; yet the nature or seat of the disease could not be ascertained. He referred his sufferings to the "inside," but he had no cough, nor fixed pain on pressure. Three months before death, he was in Bartholomew's, and was considered to be consumptive. He had there been blistered, blooded, purged, and even salivated, without the least benefit. Constipation of the bowels was obstinate, and for months before his dissolution, he never had a motion without cathartic medicine. His appetite was voracious. During the last few months of his life, a considerable tumour had gradually developed itself within the abdomen, productive of much uneasiness, but never of decided pain, although it continued to increase to the hour of his death.

*Sectio cadaveris.* Body emaciated; abdomen distended by an apparently-solid tumour. The parietes being cut through, the peritonæum was found so diseased, and so firmly adherent to the mass of intestines, that no separation or distinction of parts was practicable. The whole intestinal canal was involved in one confused mass, in which the liver, part of the stomach, and the whole of the other abdominal viscera were included. The peritonæum, where unattached, was thickened in many places to an eighth of an inch. The whole of its surface was studded with tubercles of various sizes, some as large as grapes, some smaller. In structure they resembled the yolk of a hard boiled egg, without any specific organization. An extensive heavy mass of softish disease was found between the intestines and spine, consisting of a congeries of morbid mesenteric glands, weighing fifteen or twenty pounds. All the viscera were sound in structure. In the chest water was found.

Two physicians of eminence and literary attainments, who were present, could give no name to the disease, or venture an opinion as to its nature. It was ascertained, however, some time afterwards, that the unhappy youth had been drawn into bad company, and had, in all probability, fallen a victim to depraved and *unnatural practices.* 228.

*Affections of the liver.* It is extremely difficult to decide in what diseases of this viscus *inflammation* is, or is not going on. Like the other viscera of the body, having its own peculiar structure and functions, it has also a particular measure of irritability assigned to it. The liver is sparingly supplied with nerves, and also with arterial blood; but a large stream of venous blood is constantly passing through it. When Mr. Howship was on foreign service with the army, he repeatedly found after death, inflammation and abscess of the liver, while the troops were under canvas, which he attributes to the severe colds incident to exposure during the winter season. The same observations have been made by Sir John Pringle. In civil society, occupation and artificial habits of life favour the excessive use of fermented liquors; and it is assuredly owing to this cause, that certain chronic affections of the liver are so rife among us. After relating a case of active inflammation of the liver, where mercury proved of the greatest service, Mr. Howship makes the following remark, with which we were much pleased.

"Mercury is held to be a stimulant; its effect upon the constitution is called excitement, for it certainly rouses the actions of the body, and much increases the quickness and force of the heart's action. Every case of acute inflammation is a state of local excitement, and the use of those means known to operate by stimulating, are therefore held objectionable. Upon this principle, mercury is generally recommended in chronic affections, but not in acute inflammatory action of the liver. But, in the case just related, there was not only proof of inflammatory action by the internal symptoms, but also from the local appearances. There was ground to believe suppuration was at hand, consistent with what we learn by dissection, as to the manner in which diseased parts assist themselves. There was reason to presume, that effusion and consequent adhesion had established itself between the anterior part of the liver and the adjacent parietes of the abdomen, and this carries the idea beyond mere inflammation, as it is a preparatory step to the transmission of the contents of an abscess, and perhaps very rarely, if ever, proceeds to the length marked above, unless where suppuration takes place." 288.

Case XI. *Hæmorrhage from the villous Coat of the Intestines.*—Mrs. Robinson, ætat. 42, was suddenly seized, June 1st, 1811, with severe vomiting of blood. She had previously been affected with jaundice and pain in the side. The hæmatemesis ceasing, she had frequent cough, with sickness and vomiting. Her nights were restless, with great pain down the sides of the abdomen and about the navel. She also became affected with dropsical enlargement of the abdomen.—August 10, she was seized with a fit of coughing, and threw up a considerable quantity of blood, and said she could not survive the attack. In three hours, she threw up at least a quart of blood. She had now universal tremor and faintness. She passed half a pint of blood also by stool. The sickness of stomach having left her, she remained in a very restless moaning state, with hot and dry skin, insatiable thirst, and great abdominal pain.—On the 12th, the vomiting returned, and she threw up, at intervals, two quarts of blood. General exhaustion and depression now extreme, with præcordial anxiety and low pulse; stools involuntary. The hæmatemesis ceased, but the retching continued; and in this state she remained till eight o'clock in the evening, when, sitting up in bed to change her linen, she suddenly threw up a pint of pure, thin, florid blood, which left her more reduced than ever. She died at five o'clock next morning.

*Examination.* Lower extremities anasarcous. Abdomen tumid, and about a gallon of serous fluid was found in the peritoneal cavity. Stomach healthy. Small intestines apparently natural; great intestines looked as if stained through and through with blood. The whole extent of the alimentary canal was laid open. The internal surface of the jejunum and ileum were smeared with a dark chocolate-coloured matter, apparently of blood mixed with bilious fluids. The colon and rectum were half filled with a pure blood, grumous yet fluid. The villous coat was perfect, but of a bright red or scarlet colour. No other change whatever was discernible in the intestinal track. The structure of the liver was unhealthy, and indicative of previous inflammation. The spleen was greatly enlarged, and weighed three pounds. The joint opinion of the examiners was, that the hæmorrhage had not proceeded from any one particular vessel, but evidently from the whole series of capillary arterial extremities opening on the internal surface of the intestines.

Mr. Howship was informed of a case somewhat similar, but more fortunate in its issue. The Reporter was called up in the night to a gentleman who was apparently dying from a hæmorrhage of pure blood per anum. There was no external appearance of hæmorrhoidal disease. The pulse could not be distinguished; but a pale, insensible, death-like cast of countenance. A copious injection of cold water was thrown up, and produced the happiest effects: the bowels, which previously were constantly rumbling with the discharge of blood, became completely quiet; the complaint gave way, the young gentleman recovered, and it appeared that the means used for checking the hæmorrhage had saved his life. 288.

Case XII. *Menstrual Effusion into the Cavity of the Uterus.*—With the following Case, the 97th in the work, we shall conclude our very imperfect analysis of this interesting volume.

"M. Hind, ætat. 24, was admitted into the St. George's Infirmary, December 23d, 1793, with a large tumour in the right hypogastrium, attended with excruciating pains, symptomatic fever, and a periodical discharge of menstrual blood. The pain and fever were mitigated by salines and antispasmodic fomentations, &c.; after which the infus. quercûs. was given, which restrained the menstrual discharge, and she recovered strength.

"On a more minute inquiry into the cause, duration, &c. of her complaints, she gave the following particulars: Three years before, she had become pregnant, went her full time, and was delivered of a male child. She recovered well; but a short time after, having words with a man she cohabited with, he gave her a violent blow with his foot in the lower part of the belly, just above the pubes. In consequence of this she fell ill, complaining of a violent pain in the part bruised, attended with a slight fever.

"Some medical person was called in; and in a few days, by proper medicines, the symptoms were removed, and she got perfectly well, so as to go about her usual employments as before.

"She continued well near twelve months, and was then seized with violent shivering, and severe pain in the right hypogastric region. In a day or two, she perceived an enlargement in the above-mentioned place, which continued to increase in size, as well as in pain. She now obtained admission into St. George's hospital. The tumour at this time put on the appearance of early pregnancy; but she said it was not so, for she had her menses regularly, and was at that time out of order.

"The pain, and other symptoms, were again removed by proper medicines; and in the course of two or three months the tumour had entirely subsided, and she was discharged as well.

"She remained apparently well till December 23, 1793, when she was again seized with her old complaint, only in a more violent degree than before, with slight fever, quick and small pulse, and a white tongue. The tumour had returned, and was much enlarged, occupying the whole of the right hypochondrium, and very much resembling pregnancy of the fifth month.

"She was seen by the physician, and when examined per vaginam, as there was some reason to believe she might be pregnant (except for the violent pain and discharge of her menses at the regular periods), the os uteri was readily felt, and was gently dilated. This gave discharge to a small quantity of menstrual blood, from which she found immediate ease.

"Of course she was deemed not pregnant. Medicines were ordered, and in a few weeks she was better; but the tumour did not entirely disappear. She continued tolerably well till the return of the usual period of menstruation, when she was seized with pain, &c. She had a similar relapse every returning period; and during three periods the tumour increased, and gave much pain, but not in the intervals of time. She lingered on in this state, growing weaker, till March the 7th, 1795, when a violent attack of pain in the tumour, attended with fever and severe vomiting, came on, with which she very quickly sunk and died."

*Examination.* "On opening the abdomen, the whole of the omentum was found greatly thickened from inflammation. A quantity of pus was found among the viscera; the matter, when collected, amounted to near a pint. There was universal adhesion of the intestines to each other, and to the parietes of the abdomen.

"A large tumour in the right hypochondrium, resembled very much in appearance the impregnated uterus of five months. The ovaria were both found; that on the right side perfectly healthy, and that upon the left perfectly diseased, and scirrhous.

"Considering the tumour as belonging to the uterus, a longitudinal incision was made at the fundus, where a fluctuation was perceptible, and the section gave discharge to three pounds of menstrual blood. The cyst being emptied, it was examined internally, and found to be a detached cavity formed by the external membrane of the fundus of the uterus, and having no communication with the cavity of the womb.

"There still remained a part of the tumour formed by the uterus, which was very tense, and evidently contained a fluid. A longitudinal incision was, therefore, next made along the whole course of the uterus, extending through the os uteri and vagina. This gave discharge to one pound more of menstrual blood. The uterus, now quite flaccid and empty, the internal part was examined, to see if there was any communication with the aforesaid cavity, but none was discovered.

"The uterus was slightly scirrhous in its texture, and tuberculated. In the inside of the uterus there were many blind pouches, formed by duplicatures of the internal membrane.

"The openings of the Fallopian tubes into the uterus were much enlarged.

"The bladder was very much contracted, and adherent from inflammation to the uterus; but this circumstance had not been productive of any retention of urine." p. 357.

Notwithstanding the extent of our analysis, such is the immense mass of practical facts and information contained in Mr. Howship's work, that we have not been able to notice more than a tenth part of its contents. After this declaration, and the specimens which we have brought forward, it is hardly necessary to pay the work a higher compliment, or offer a greater inducement to every medical practitioner to peruse the original, and place it in his library for constant reference.

